# A Popular Drug

**Published:** 1902-09-27

**Authors:** 


### A POPULAR DRUG.

Commenting upon the perennial popularity of nux vomica as a tonic the *Polyclinic* says that there is probably no single compound concerning which patients are more unanimous that they feel better when they take it than they are in regard to Easton's syrup, and that the popularity of this mixture is probably due to the strychnia which it contains. A successful physician of the past century used to advise his pupils to put tincture of nux vomica into almost all their prescriptions. "Your patient may perhaps not be cured of his special ailment, but you will always be told that your mixture has improved his general health. Almost everybody is the better for a tonic, and nux vomica is the best of tonics. It agrees with everybody." Were that physician living he would be delighted to observe the repute which his favourite drug has gained. From Martindale's list it appears that this tincture is prescribed more frequently than any other drug, standing ahead even of such generally employed adjuncts as chloric ether and sal volatile. It is a far safer drug for general use than any preparation of strychnia. Whilst five drops is a very efficient tonic, thirty may be taken without serious consequences.

